# Ovotransferrin exerts bidirectional immunomodulatory activities via TLR4‐mediated signal transduction pathways in RAW264.7 cells

**DOI:** 10.1002/fsn3.2569

**Published:** 2021-09-08

**Authors:** Zhiying Ru, Mingsheng Xu, Gaoxiang Zhu, Yonggang Tu, Yan Jiang, Huaying Du

**Affiliations:** ^1^ Key Laboratory of Natural Product and Functional Food of Jiangxi Jiangxi Agricultural University Nanchang China

**Keywords:** inflammation, MyD88, ovotransferrin, RAW 264.7 macrophage, toll‐like receptor 4

## Abstract

The immune regulation function of ovotransferrin (OVT) explored using the RAW264.7 was induced by lipopolysaccharide (LPS) as vitro model in this study. The results showed that RAW264.7 cultured with OVT (200 μg/ml) alone not only enhanced the phagocytic activity and the production and expression of inflammatory factors, but also expression of toll‐like receptor 4 (TLR4) gene was significantly promoted by OVT. OVT (50 μg/ml) significantly inhibited the secretion and expression of inflammatory factors in LPS‐stimulated RAW264.7, but CD14 and TLR4 genes expressions were no obvious effects. Inflammatory cytokines and NO secreted by OVT‐induced macrophages pretreated with inhibitors of TLR4 were down‐regulated. We further verified the effects of OVT on inflammatory signaling pathway‐related proteins through immunofluorescence and western blotting, MyD88, TLR4 and the phosphorylation of IκBα and p65 were significantly promoted by OVT, but there was no significant effects on the phosphorylation of IRF3. OVT promoted the phosphorylation of ERK and p38 in RAW264.7 and inhibited the phosphorylated expression of MAPK in LPS‐mediated inflammation. These results indicated that OVT had the bidirectional immunoregulatory function through TLR4‐mediated NF‐κB/MAPK signaling pathway, that is, anti‐inflammatory effect of low concentration and immune‐enhancing activity of high concentration were showed. That provides a theoretical utilization for the development and utilization of OVT.

## Graphical abstract

Ovotransferrin had the bidirectional immunoregulatory function through TLR4‐mediated NF‐κB/MAPK signaling pathway, that is, anti‐inflammatory effect of low concentration and immune‐enhancing activity of high concentration were showed.

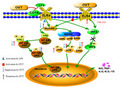

## 1 INTRODUCTION

Many foods not only provide the body with essential nutrients and trace elements every day, but also play an important role in the health of the body, such as immune and metabolic regulation (Pang et al., 2006). Poultry eggs are the main food for human, providing many nutriment, such as protein, fat and vitamins. Egg white comprises 60% of the entire poultry egg content and contains ovalbumin, ovotransferrin, ovomucoid, ovomucin and lysozyme (Mine, 2002). Ovotransferrin (OVT) is one of the major functional proteins, accounting for approximately 12%–13% of total egg white content (Wu & Acero‐Lopez, 2012). At the same time, it is the acidic soluble and good iron‐binding glycoprotein consisting of 686 amino acid in the poultry transferrin family. In addition to its excellent immunoregulatory activity, OVT is also involved in antigen recognition and initiation of immune response. Furthermore, previous studies have shown that OVT currently plays an obvious role in antibacterial (Schade & Caroline, 1944), antiviral (Giansanti et al., 2002), antioxidant (Majumder et al., 2013) and immune‐enhancing activity (Lee et al., 2018). There is still an increasing interest in the search for the immunity regulation of OVT.

Ovotransferrin is a component of natural immunity (Shang & Wu, 2018). Likewise, it participates in a series of immune regulation response. OVT, initially as an avian acute phase protein (Rath, 2005), is reported to modulate macrophage and heterophil function by stimulating the production of interleukin‐6 (IL‐6) and nitrite (Xie et al., 2002) and promote the proliferation of splenic lymphocytes (Xu, Lin, et al., 2012). In addition, oral administration of OVT reduces the secretion of inflammatory cytokines and slow the spread of the colitis induced by dextran sodium sulfate (Kobayashi et al., 2015) and enhances immune response in cyclophosphamide‐induced immunosuppressed mice (Zhu et al., 2018). This suggests that OVT may have bidirectional immunomodulatory effects in vivo. Our previous research finds that OVT increases the secretion of TNF‐α though MAPK signaling pathway (Liu et al., 2017) in DCs. More recently, OVT exerts in vitro immune activity by MAPK signaling pathway. These findings are based on inflammation models, but how OVT stimulates macrophages to exert immunomodulatory effects is currently less researched in noninflammatory models. In addition, there are few studies on whether OVT can play an anti‐inflammatory role in LPS‐induced inflammation by inhibiting the expression of signaling pathway‐related proteins.

The immunomodulatory effect of protein may be mediated by the direct binding of food‐derived proteins to immune cell surface receptors, thereby activating related signaling pathways mediated by cell surface receptors on the one hand (Yang et al., 2019). On the other hand, the binding of LPS and the plasma membrane CD14 protein, a GPI‐anchored membrane protein in bone marrow cells, consults macrophage activation. LPS is next transferred to TLR4 triggering signal transduction (Prymas et al., 2020), which begins to recruit a single or specific receptor combination in the TIR domain containing the receptor protein. Then, NF‐κB was activated by almost all known immune cell surface receptors (Oeckinghaus et al., 2011) and MAPK pathways including p38, JNK and ERK are stimulated. It is known that immunomodulatory activity of most food‐derived protein is displayed by inhibiting the signal components of NF‐κB or MAPK pathway (Han et al., 2009; Kim et al., 2016).

Therefore, the purpose of the present study was to investigate the receptor to which OVT binds directly and whether OVT modulates MyD88‐dependent and MyD88‐independent, namely TRIF signaling pathways. Here, we determined that TLR4 may be the membrane receptor protein of OVT on the expression level of TLR1, TLR2 and TLR4. In addition, our results found that OVT regulated inflammation by TLR4/MyD88/NF‐κB/MAPK inflammatory signaling using western blot. To our knowledge, it is reported for the first time that the TLR4 may be involved in the OVT binding on RAW 264.7 cells to trigger immune‐enhancing response. That provides theoretical basis for the development of OVT as immunomodulator and enhancer.

## 2 MATERIALS AND METHODS

### 2.1 Materials and chemicals

Dulbecco's Modified Eagle's Medium (DMEM) and fetal bovine serum (FBS) were obtained from Biological Industries (Bioind). Penicillin–streptomycin was purchased from Gibco BRL Co. Ltd. Ovotransferrin from egg white, purity of which is over 98%, and LPS (from *Escherichia coli*, 0111:B4) were purchased from Sigma‐Aldrich. Mouse Enzyme‐Linked Immune‐Sorbent Assay (ELISA) kits were purchased from Thermo Fisher Scientific. Hoechst 33258 fluorescent dye (DAPI) was purchased from Bioworld Biotechnology CO., Ltd. CCK assay kit was obtained from TransGen Biotech Company. TAK‐242 inhibitor was purchased from MedChem Express. All associated antibodies except TLR4 were purchased from Cell Signaling Technology. TLR4 antibody, secondary antibodies and chemiluminescence (ECL) detection kit were purchased from ProteinTech Group, Inc.

### 2.2 Cell culture

RAW264.7 cells were purchased from Procell Life Science Technology Co, Ltd, which were cultured in DEME high glucose medium containing 10% FBS and 1% penicillin and streptomycin and incubated in an incubator at 37°C in humidified 5% CO_2_ atmosphere. The logarithmic growth phase cells are selected for subsequent seed plate experiments.

### 2.3 Cell viability assay

The CCK assay (Li, Sun, et al., 2019) was used for determining effect of OVT with various concentrations on the viability of cell. Two hundred microliter of cell suspension with a density of 1 × 10^5^ cells was added to the 96‐well plate. The control group was added with complete culture medium; the LPS model group was added with 100 ng/ml LPS solution; the OVT treatment group was added with mixed solutions containing LPS (100 ng/ml) and OVT of different concentrations; and the OVT control group was added with OVT of different concentrations, and the cells were furfure cultured for 24 h. Subsequently, 10 µl CCK solution was added and incubated for 2 h. Absorbance value was estimated at 450 nm by a microplate reader. Cell viability was calculated by the following equation.

$$\text{Cell}\;\text{activity}\;\left(\%\right)=\frac{A\;\left(\text{treated}\right)‐A\;\left(\text{blank}\right)}{A\;\left(\text{control}\right)‐A\;\left(\text{blank}\right)}\times 100$$

*A* (treated): OD of wells including cells and OVT solution.

*A* (blank): OD of wells including complete culture solution only.

*A* (control): OD of wells including cells and complete culture solution.

### 2.4 Phagocytosis of neutral red assay

Macrophages phagocytosis was detected using neutral red uptake assay from Beijing solarbio science & technology co., Ltd (Huang et al., 2019). The cells were grouped and cultured according to the previous experiment. After incubation for 24 h, cells were cultured with 200 µl complete medium and 20 µl neutral red solution for a further 3 h incubation. PBS was used to wash cells for twice times to remove medium containing neutral red, and then, 200 µl neutral red detection lysis solution was added. The cells were lysed on the shaker at room temperature for 10 min to promote cell lysis in sync. The absorbance values at 560 nm were acquired.

### 2.5 Measurement of nitric oxide (NO)

The content of NO was assessed using Griess assay (Xiong et al., 2018) to indirectly reflect the degree of inflammation. The cells were grouped and cultured according to the previous experiment. The concentration of NO was measured by using NO assay kit (Beyotime Biotechnology Co., Ltd). Culture supernatant (50 µl) and 100 µl Griess solution (Griess A:Griess B = 1:1) were added to a 96‐well plate. Take care to avoid light and measure the absorbance at 540 nm using a microplate reader.

### 2.6 Measurement of cytokine production

RAW 264.7 cells with a density of 1 × 10^6^ cells/ml were seeded onto 24‐well plates and incubated for 6 h. The supernatant was discarded. The control group was added with complete culture medium; the LPS model group was added with LPS solution containing 100 ng/ml LPS; the OVT treatment group was added with mixed solutions containing LPS (100 ng/ml) and OVT (50, 100 and 200 μg/ml); and the OVT control group was added with OVT at 50, 100 and 200 μg/ml and the cells were cultured for 24 h. And the supernatant was collected and centrifugal. Levels of TNF‐α, IL‐6 and IL‐10 in the supernatant were measured using a microplate reader at 450 nm according to the manufacturer's instructions.

### 2.7 Real‐time quantitative reverse transcription PCR (qRT‐PCR)

Cells were seeded in a 6‐well plate at 1 × 10^6^ cells/ml, cultured for 6 h and then adhered to the wall for group treatment. Total RNA was collected from the RAW264.7 cells using the RNA extraction kit from TransGen Biotech Company. To ensure that subsequent experiments can be carried out, the BioDrop µLife+ (BioDrop) is needed to detect purity and integrity. Total RNA (2 µg) was revamped into cDNA by cDNA Synthesis SuperMix kit. The PCR amplification of cDNA was performed with specific primers for TNF‐α, IL‐6, IL‐10, CD14, TLR1, TLR2, TLR4 and β‐actin as the control. The forward and reverse primers sequences which are designed and synthesized are listed in Table 1. Amplification conditions were as follows: 94°C initial denaturation for 30 s followed by 42 cycle of 94°C for 5 s and 62°C for 30 s. Relative expression levels of the target genes were calculated using 2^−ΔΔCt^.

**TABLE 1:** Primers used in this study

Gene	Forward primer (5′−3′)	Reverse primer (5′−3′)
β‐actin	CCACAGCTGAGAGGGAAATC	TCTCCAGGGAGGAAGAGGAT
TNF‐α	CTGGGACAGTGACCTGGACT	GCACCTCAGGGAAGAGTCTG
IL‐6	GACTGATGCTGGTGACAACC	AGACAGGTCTGTTGGGAGTG
IL‐10	TAACTGCACCCACTTCCCAG	AAGGCTTGGCAACCCAAGTA
CD14	TTCTGAGGGTCCTCGTCAAC	CGTGTGGATCCTGAGGGTTA
TLR1	TCATTGTCCAAGCTGAGGGT	GCAGGGCATCAAAGGCATTA
TLR2	TCTAAAGTCGATCCGCGACA	ATCTACGGGCAGTGGTGAAA
TLR4	TAGCCATTGCTGCCAACATC	CCTCAGCAGGGACTTCTCAA

### 2.8 Immunofluorescence assay

Lipopolysaccharide is known to activate NF‐κB, which regulates the inflammatory response by modulating multiple proinflammatory cytokines in macrophages (Ren et al., 2020). To further investigate whether the anti‐inflammatory effects of OVT are mediated by the NF‐κB signaling pathway, p65 NF‐κB expression in macrophage nucleus was observed by the immunofluorescence in this study. Cells (2 × 10^5^/ml) were set in 24‐well plates and treated with 200 µg/ml OVT, LPS and LPS+OVT for 24 h. After being fixed with 4% paraformaldehyde for 30 min at room temperature, penetrated with 0.5% Trixon‐100(prepared by PBS) and blocked with 2% of BSA, the cells were incubated with primary anti‐NF‐κB p65 antibody (1:500) overnight at 4°C, followed by the secondary antibody (labeled FITC) in dark for 2 h. And cells were stained with Hoechst 33258 fluorescent dye (DAPI) for 20 min. The photos were collected by inverted fluorescence microscopy (OLYMPUS; 20×).

### 2.9 Western blot analysis of protein expression

Cells were seeded in a 6‐well plate at 1 × 10^6^ cells/ml, cultured for 6 h and then adhered to the wall for group treatment. Groups are divided into control, OVT (200 µg/ml), LPS and LPS+OVT (50 µg/ml) and further cultured for 24 h. Radioimmunoprecipitation assay (RIPA) buffer (1% EDTA, protease and phosphatase inhibitor) was used to extract cellular protein for 15 min. The concentration of cellular protein was determined with BCA protein assay kit from Beijing solarbio science & technology co., Ltd, and uniformly adjusted to 2.5 mg/ml. Loading buffer was added into proteins (v:v = 1:5) to make it denatured by boiling at 100°C for 5 min. Denatured protein was separated by 10% SDS‐polyacrylamide gel electrophoresis (SDS‐PAGE) and transferred to a 0.45 µm PVDF (Millipore) at a transferred membrane condition of 200 mA and 80 min. PVDF membrane is trimmed according to molecular weight. After sealing with 5% BSA for 1.5 h, the membranes were overnight incubated with the following antibodies (1:1000 dilution): TLR4, MyD88, IRF3, p38, p‐p38, ERK, p‐ERK, JNK, p‐JNK, IκBα, p‐IκBα, p65, p‐p65 at 4°C. They were incubated with secondary antibodies for 1 h at room temperature. The antibody‐specific protein was viewed by enhanced chemiluminescent detection system with ECL kit.

### 2.10 TLR4 inhibition assay in OVT‐stimulated RAW264.7

RAW 264.7 cells with a density of 1 × 10^6^ cells/ml were seeded onto 24‐well plates and incubated for 6 h. The supernatant was discarded. The control group was added with complete culture medium, the OVT group was added with 200 μg/ml OVT, and theTAK‐242 group was added with 50 nM TAK‐242. And the cells were pretreated with TAK‐242(50 nM) for 8 h prior to the addition of 200 μg/ml OVT for 24 h. And the supernatant was collected and centrifugal. Levels of NO and TNF‐α in the supernatant were measured using a microplate reader at 450 nm according to the manufacturer's instructions.

### 2.11 Statistical analysis

Data are showed as means ± standard error of the mean (*SEM*) and statistical significant analysis is determined by Duncan's test with a *p* < .05 taken as value of significance. All the figures were drawn using the GraphPad Prism 7.0 software.

## 3 RESULTS

### 3.1 No inhibitory effect of OVT on the cell viability of RAW264.7

**FIGURE 1:**
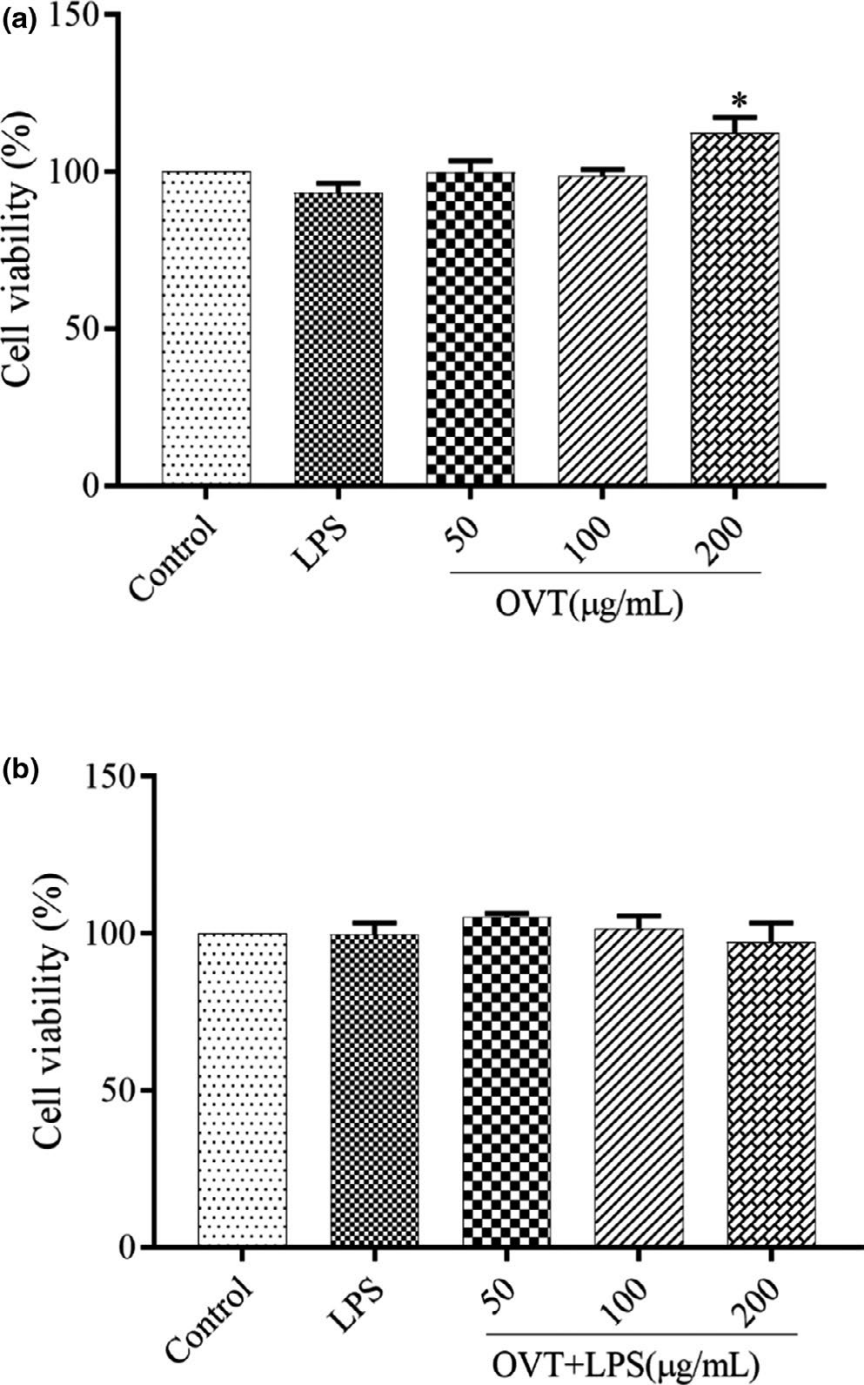
The viability of RAW264.7 macrophages cultured for 24 h under the conditions of OVT. Data are shown as means ± *SEM* (*n* = 6). (a) Compared with the control group: *p < .05, **p < .01; (b) compared with the LPS group: ^#^ *p* < .05, ^##^ *p* < .01

CCK assay is used to detect whether OVT with various concentrations has damage to cell viability (Li, Sun, et al., 2019). CCK is a reagent for cell proliferation and cytotoxicity detection based on water‐soluble tetrazolium salt. In the presence of electron coupling reagent 1‐methoxy PMS, it can be reduced to soluble orange‐yellow formazan by dehydrogenase in mitochondria, and the shade of color can directly reflect whether the protein is toxic to cells. As shown in Figure 1a, within a given concentration range, cellular activity was slightly increased in the OVT‐stimulated RAW264.7. At the condition of OVT concentration of 200 µg/ml, the cell activity reached the maximum of 112 ± 8%. The cellular activity was found no effect in LPS‐stimulated RAW264.7 treated OVT (Figure 1b). These results indicated that OVT had no toxicity to macrophages and the concentration conditions could be used in subsequent experiments.

### 3.2 OVT promoted phagocytosis activity of RAW264.7 cells and inhibited phagocytosis activity of LPS‐stimulated RAW264.7

**FIGURE 2:**
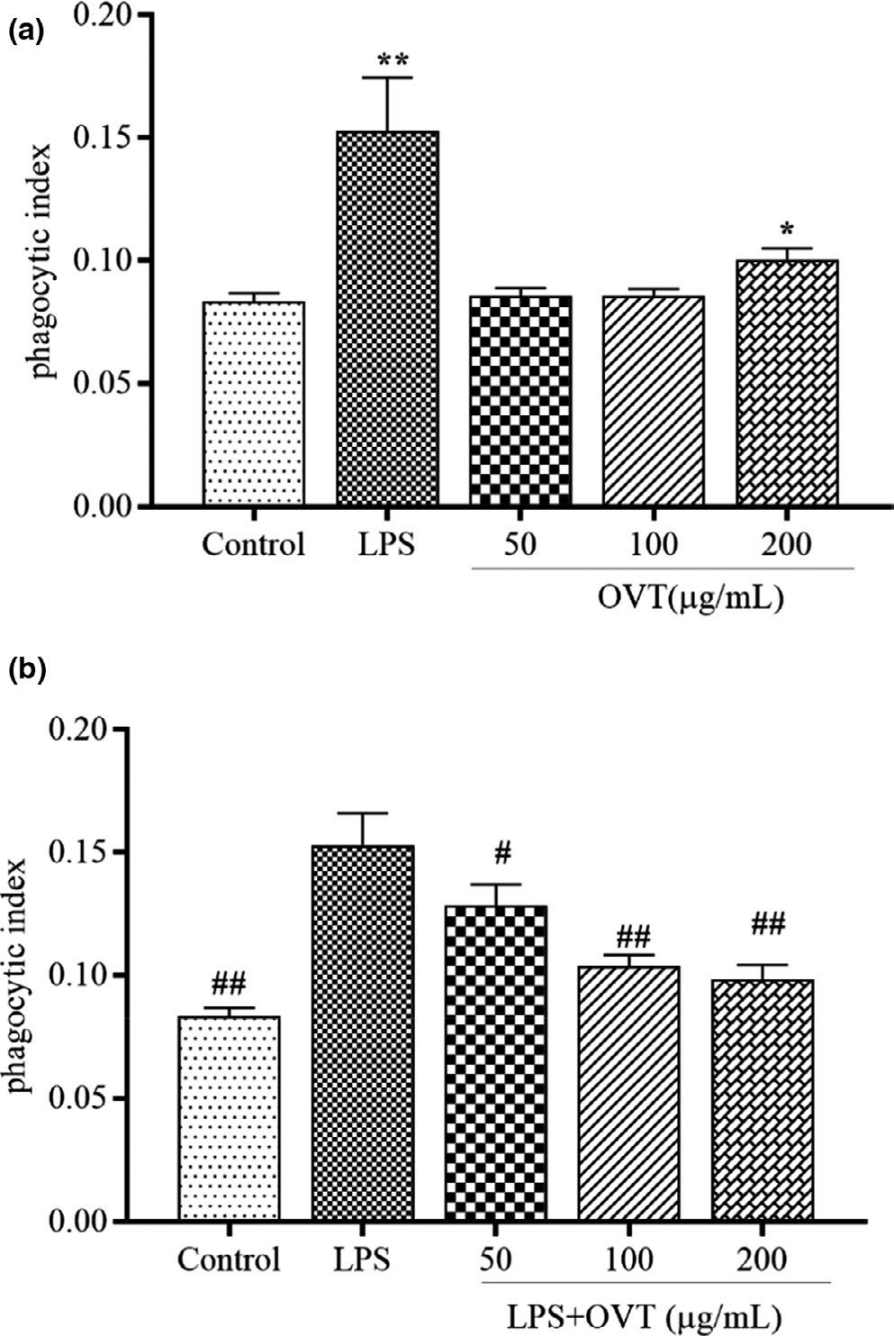
(a) Promoting effect of OVT on phagocytosis activity of RAW264.7 cells and (b) inhibitory effect of LPS‐stimulated RAW264.7 treated with OVT for 24 h using neutral red assay. Data are shown as means ± *SEM* (*n* = 4). Compared with the control group: *p < .05, **p < .01; compared with the LPS group: ^#^ *p* < .05, ^##^ *p* < .01

When pathological changes occur in tissues and organs, macrophages can eliminate antigens by phagocytosis (Xiong et al., 2018). Neutral red phagocytosis experiment is commonly applied to evaluate the phagocytic activities of RAW264.7 cells treated with various concentrations of OVT (Wang et al., 2010). Cell phagocytosis was promoted slightly by OVT compared with the normal control group (*p* > .05) and increasing effect of 200 µg/ml OVT treatment group was significant (*p* < .05; Figure 2a). In the LPS‐stimulated RAW264.7, the phagocytic rate of cells was significantly inhibited of OVT in Figure 2b (*p* < .05). As the concentration increases, the stimulating effect of OVT on the phagocytic ability of RAW264.7 cells was also continuously decreasing.

### 3.3 OVT promoted production of NO in RAW264.7 cells and inhibited production of NO in LPS‐stimulated RAW264.7

**FIGURE 3:**
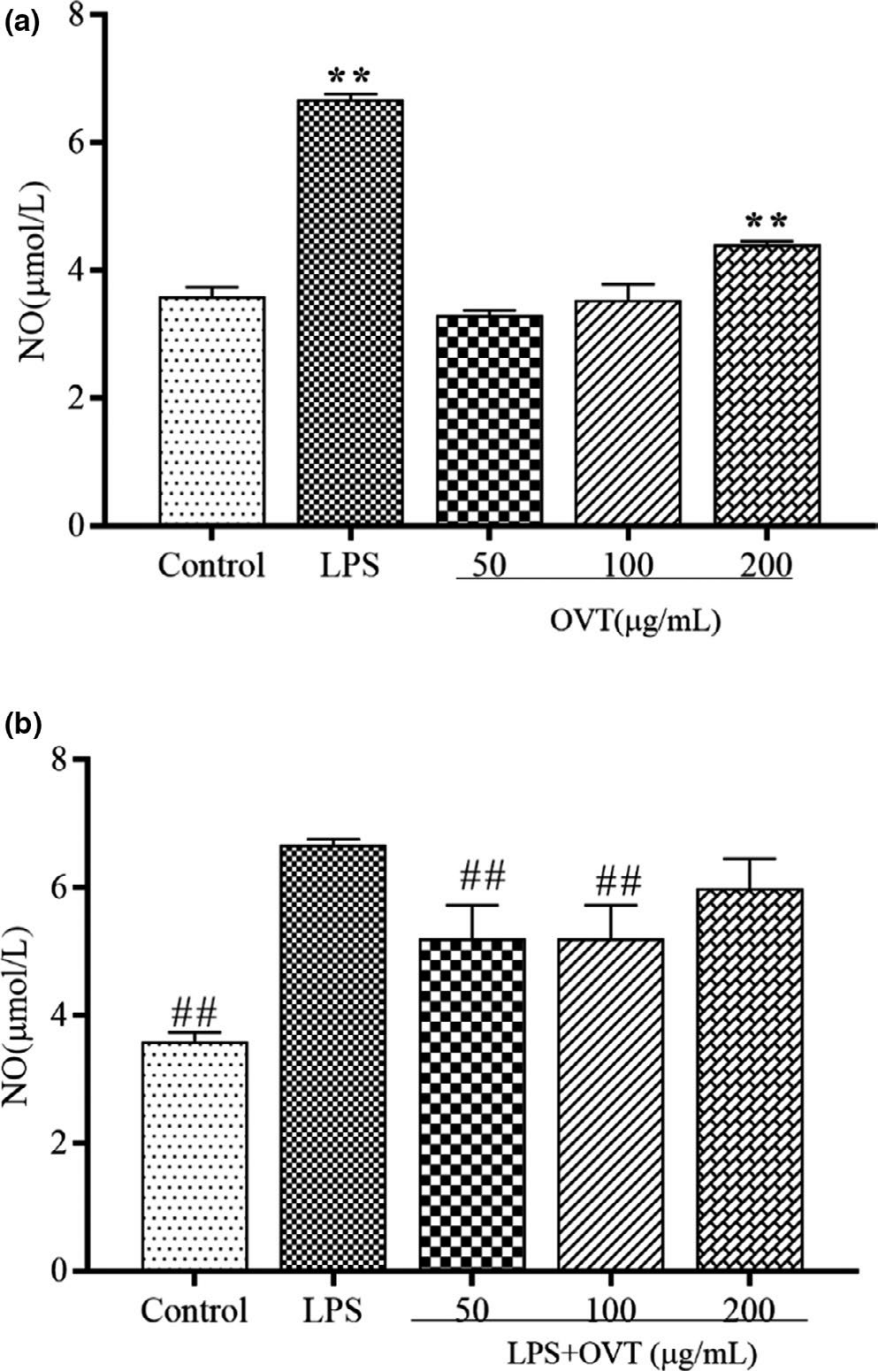
(a) Nitric oxide (NO) production promotion in OVT‐induced RAW264.7 and (b) NO production inhibition of OVT in LPS‐stimulated RAW264.7. Macrophages were incubated with various concentrations of OVT in the presence or absence of LPS (100 ng/ml) for 24 h. NO level in culture media was displayed using Griess assay. Data are shown as means ± *SEM* (*n* = 6). Compared with the control group: *p < .05, **p < .01; compared with the LPS group: ^#^ *p* < .05, ^##^ *p* < .01

NO, an important transmembrane molecular signal, positively reflects the severity of inflammation in the inflammatory process, and it is also a basic condition for macrophages to play phagocytic function (Huang et al., 2014; Ye et al., 2020). To confirm the extent of the inflammatory response to OVT, the amount of NO produced was analyzed. As shown in Figure 3a, as expected, a minimum amount of NO was produced in control group; while LPS‐stimulated macrophages released NO at a level of 6.68 ± 0.15 μM. NO production is promoted in a dose‐dependent manner in OVT‐induced RAW264.7. Among them, the production of NO treated with OVT at 200 µg/ml was higher than those in other groups, but it was lower than LPS group (*p* < .01). As shown in Figure 3b, compared with the LPS group, the treatment of OVT (50 µg/ml) significantly reduced the levels of NO in LPS‐induced macrophages (*p* < .01). In addition, the production of NO in the RAW264.7 cocultured with LPS and OVT was higher than that of OVT‐induced RAW264.7 macrophages only, indicating that OVT and LPS had a synergistic effect on NO production.

### 3.4 OVT promoted release of TNF‐α, IL‐6 and IL‐10 in RAW264.7 cells and inhibited release of these cytokines in LPS‐stimulated RAW264.7

**FIGURE 4:**
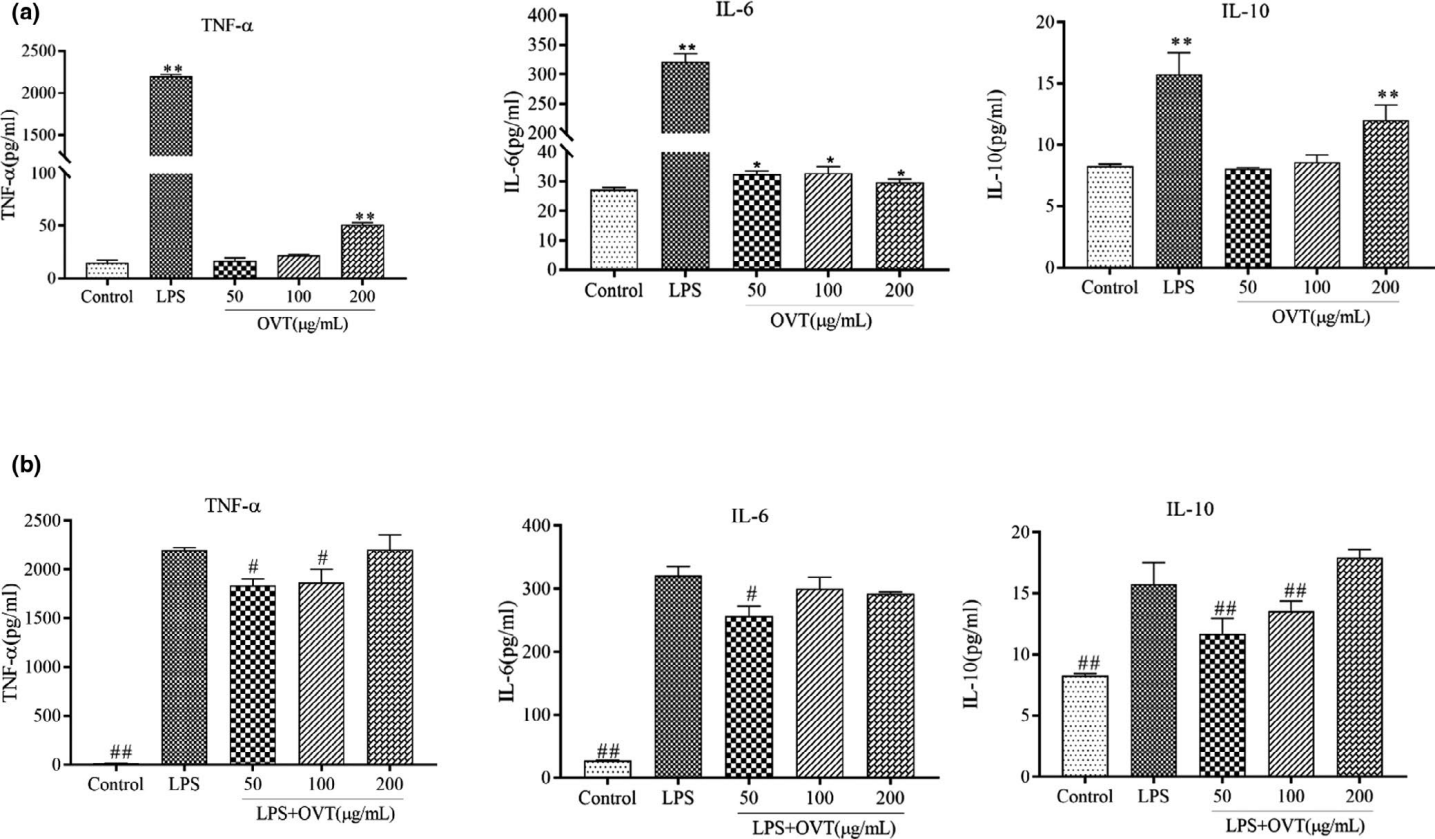
(a) Promoting effect of OVT on release of TNF‐α, IL‐6 and IL‐10 in RAW264.7 cells and (b) inhibitory effect of OVT on LPS‐stimulated release of TNF‐α, IL‐6 and IL‐10. Macrophages were incubated with various concentrations of OVT in the presence or absence of LPS (100 ng/ml) for 24 h. The TNF‐α, IL‐6 and IL‐10 levels in the cell culture media were measured by ELISA. Data are shown as means ± *SEM* (*n* = 3). (a) Compared with the control group: *p < .05, **p < .01; (b) compared with the LPS group: ^#^ *p* < .05, ^##^ *p* < .01

Macrophages meditate immune response through secreting the inflammatory cytokines (tumor necrosis factor‐α [TNF‐α], IL‐6) and anti‐inflammatory cytokines (interleukin‐10 [IL‐10]; Lai et al., 2020; Liu et al., 2017; Shapouri‐Moghaddam et al., 2018). In consequence, to examine the strength of the immunoregulatory activity of OVT, the amounts of OVT on the IL‐6, TNF‐α and IL‐10 secretion in RAW264.7 and LPS‐induced RAW264.7 by ELISA are represented in Figure 4. Figure 4a shows the effect of OVT on the cytokines (TNF‐α, IL‐6 and IL‐10) production in normal RAW 264.7 macrophages. After LPS treatment (100 ng/ml), the expression of TNF‐α, IL‐6 and IL‐10 was dramatically increased (*p* < .01) compared with control group. The effect of OVT on cytokine secretion in RAW264.7 cells was correlated with the concentration. The secretion of TNF‐α, IL‐6 and IL‐10 had significantly raised, respectively (*p* < .01) by 271%, 21%, 46% in OVT (200 μg/ml) stimulation group (Figure 4a). As shown in Figure 4b, OVT (50 and 100 µg/ml) had a significant inhibitory effect in LPS‐stimulated RAW264.7 cells (*p* < .05). OVT inhibited, respectively, TNF‐α, IL‐6 and IL‐10 production by 16%, 19% and 25% at concentration of 50 µg/ml, while the 200 µg/ml OVT resulted in no inhibition of cytokines production. The release of TNF‐α, IL‐6 and IL‐10 in the RAW264.7 cocultured with LPS and OVT was higher than that of OVT‐induced RAW264.7 macrophages only. In the meanwhile, the results showed that OVT and LPS had a collegial effect on the release of TNF‐α, IL‐6 and IL‐10.

### 3.5 OVT promoted expression of TNF‐α, IL‐6 and IL‐10 gene in RAW264.7 and inhibited expression of these gene in LPS‐stimulated RAW264.7

**FIGURE 5:**
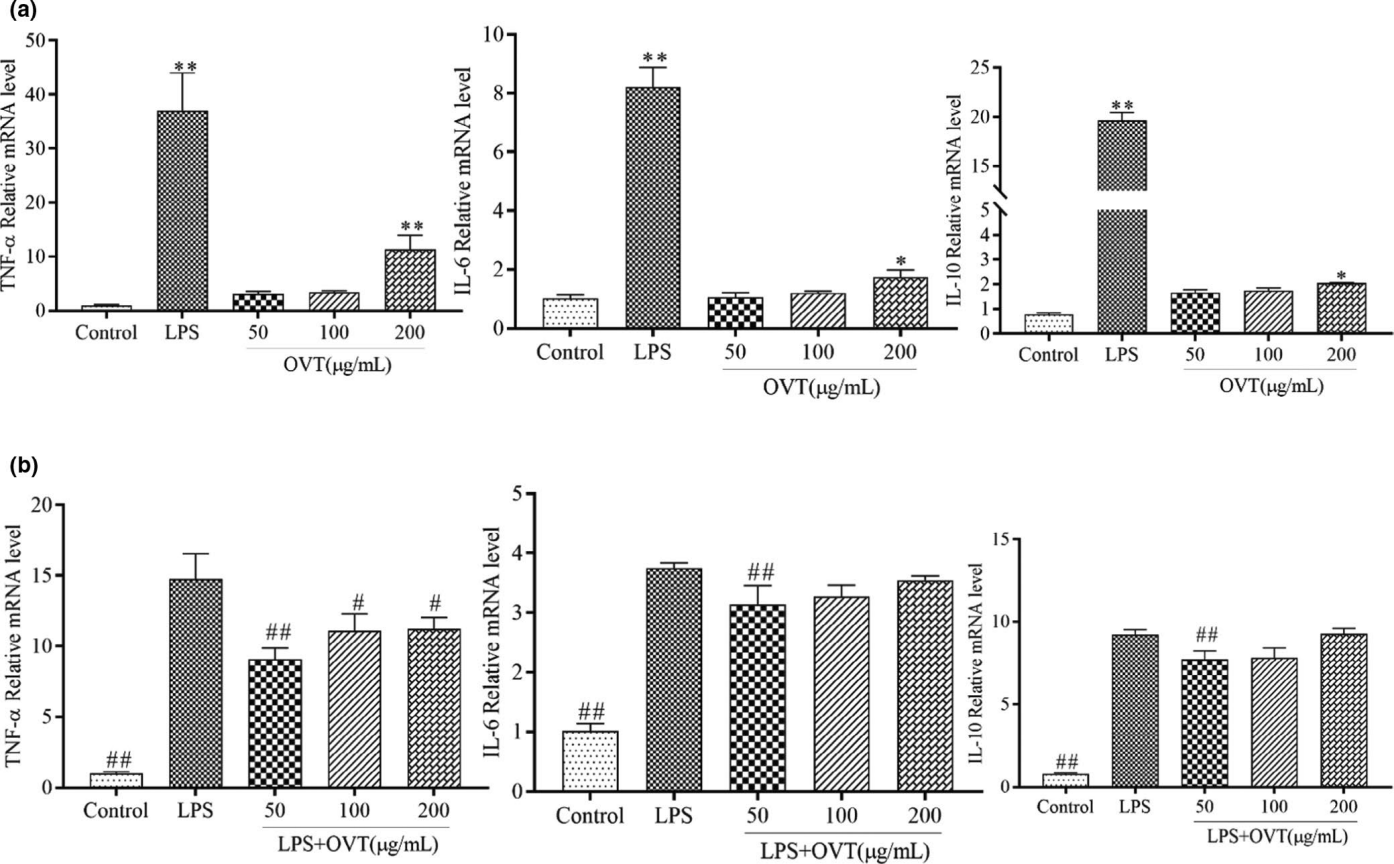
(a) Promoting effect of OVT on mRNA expression of TNF‐α, IL‐6 and IL‐10 in RAW264.7 cells and (b) inhibitory effect of OVT on LPS‐stimulated mRNA expression of TNF‐α, IL‐6 and IL‐10. Macrophages were incubated with various concentrations of OVT in the presence or absence of LPS (100 ng/ml) for 24 h. Expression of these genes of cell lysates was assessed by RT‐PCR. Data are shown as means ± *SEM* (*n* = 3). (a) Compared with the control group: *p < .05, **p < .01; (b) compared with the LPS group: ^#^ *p* < .05, ^##^ *p* < .01

To further determine whether OVT controls changes in inflammatory factors by regulating gene expression, we examined TNF‐α, IL‐6 and IL‐10 gene expressions in OVT‐stimulated RAW264.7 cells and LPS‐stimulated RAW264.7 treated with OVT. As shown in Figure 5a, increased gene expressions of TNF‐α, IL‐6 and IL‐10 were observed by OVT treatment in a concentration‐dependent manner in RAW264.7 cells. OVT (50 and 100 µg/ml) significantly inhibited the expression of TNF‐α. IL‐6 and IL‐10 of LPS‐stimulated RAW264.7 (*p* < .05; Figure 5b), which was consistent with the secretion of TNF‐α, IL‐6 and IL‐10. Collectively, these data further corroborated that production of TNF‐α, IL‐6 and IL‐10 was promoted by OVT in the RAW264.7 cells by up‐regulating their gene expression.

### 3.6 OVT improved TLR4 expression in RAW264.7 and slightly enhanced expression of these gene in LPS‐stimulated RAW264.7

**FIGURE 6:**
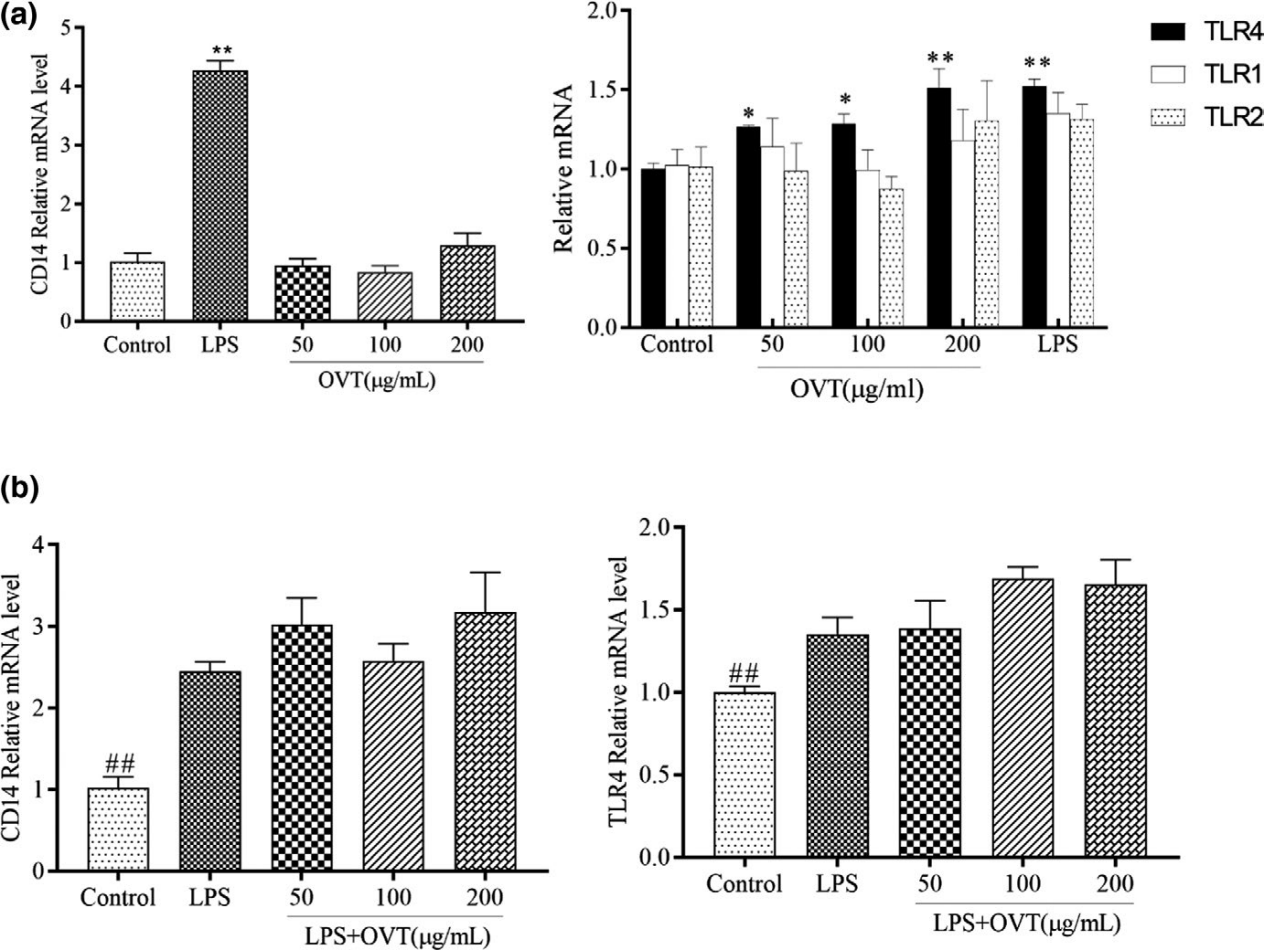
(a) Promoting effect of OVT on mRNA expression of TLR4 in RAW264.7 cells and no effect on mRNA expression of CD14, TLR1 and TLR2 and (b) no effect of OVT on LPS‐stimulated mRNA expression of CD14 and TLR4. Macrophages were incubated with various concentrations of OVT in the presence or absence of LPS (100 ng/ml) for 24 h. Expression of these genes of cell lysates was assessed by RT‐PCR. Data are shown as means ± *SEM* (*n* = 3). (a) Compared with the control group: *p < .05, **p < .01; (b) compared with the LPS group: ^#^ *p* < .05, ^##^ *p* < .01

CD14 shares the binding site of LPS, effectively transfers LPS to TLR4 and mediates the production of proinflammatory factors and nitric oxide (Yu et al., 2014). TLR2 is the molecule with the most extensive expression range among members of the TLRs family, participating in inflammatory signal transduction and mediates natural antiinfection immunity. The TLR1/TLR2 combination can recognize bacterial lipoproteins (Kawasaki & Kawai, 2014). In order to examine the change of CD14 and the membrane receptor of OVT, expression level of CD14, TLR1, TLR2 and TLR4 based on RT‐PCR was measured. Figure 6a reflects that the expression levels of CD14 and TLR4 receptors in the LPS group were dramatically increased (*p* < .01). Expression of TLR4 is in a concentration‐dependent manner at the transcriptional level (Figure 6a; *p* < .05 at 50 and 100 μg/ml; *p* < .01 at 200 μg/ml) in OVT‐stimulated RAW264.7 cells, with no effect on the CD14, TLR1 and TLR2 receptors (*p* > .05) in normal RAW264.7 cells. OVT enhanced the expression levels of CD14 and TLR4 receptors in LPS‐induced macrophages (*p* > .05; Figure 6b). Therefore, OVT may enter macrophages through TLR4 receptors in vitro to activate immunomodulatory effects, not by inhibiting receptor gene expression to exert anti‐inflammatory effects.

### 3.7 Effect of OVT on transfer of p65 NF‐κB proteins into nucleus

**FIGURE 7:**
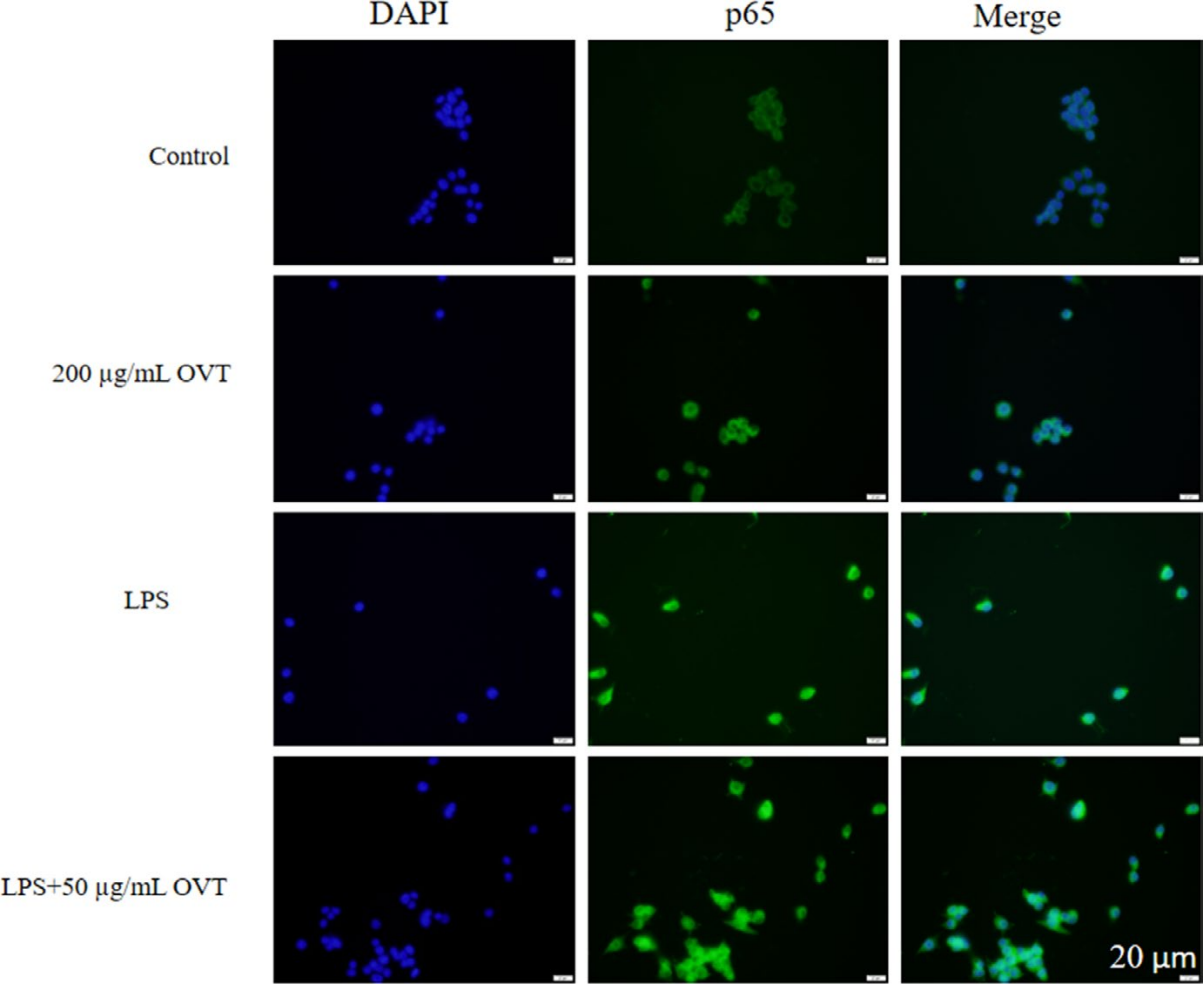
Effect of OVT on immunofluorescence subcellular localization of p65 protein in LPS‐stimulated RAW264.7 cells. RAW264.7 cells were treated with OVT (200 µg/ml) and cotreated with LPS and OVT (50 µg/ml) for 24 h, p65 protein localization was immunochemically detected using anti‐p65 antibody. The same fields were stained with Hoechst 33258 for the location of nuclear

Activation of NF‐κB was confirmed by immunofluorescence staining to determine whether p65, the major subunit of NF‐κB, was transported to the nucleus. In the resting state, NF‐κB dimer combined with the inhibitory subunit IκB to form the p65‐p50‐IκB trimer complex. When cells are stimulated by activation factors such as inflammatory factors, IκB is phosphorylated, degraded and dissociated from the complex, exposing the nuclear signaling region, and transferring from the cytoplasm into the nucleus to bind to the IκB site on the target motif to initiate transcription. Fluorescence staining results are shown in Figure 7. NF‐κB p65 in the blank control group was mainly located in the cytoplasm. After 200 μg/ml OVT treatment, part of p65 migrated to the nucleus, and the cytoplasm and nucleus of the LPS group showed bright green light, indicating that the p65 protein in the nucleus was significantly increased, and low concentrations of OVT inhibited the migration of p65. The results showed that OVT could regulate the expression of NF‐κB through nuclear translocation of p65.

### 3.8 OVT promoted protein expression of TLR4, MyD88, NF‐κB and MAPK in RAW264.7 cells and inhibited expression of these proteins in LPS‐stimulated RAW264.7

**FIGURE 8:**
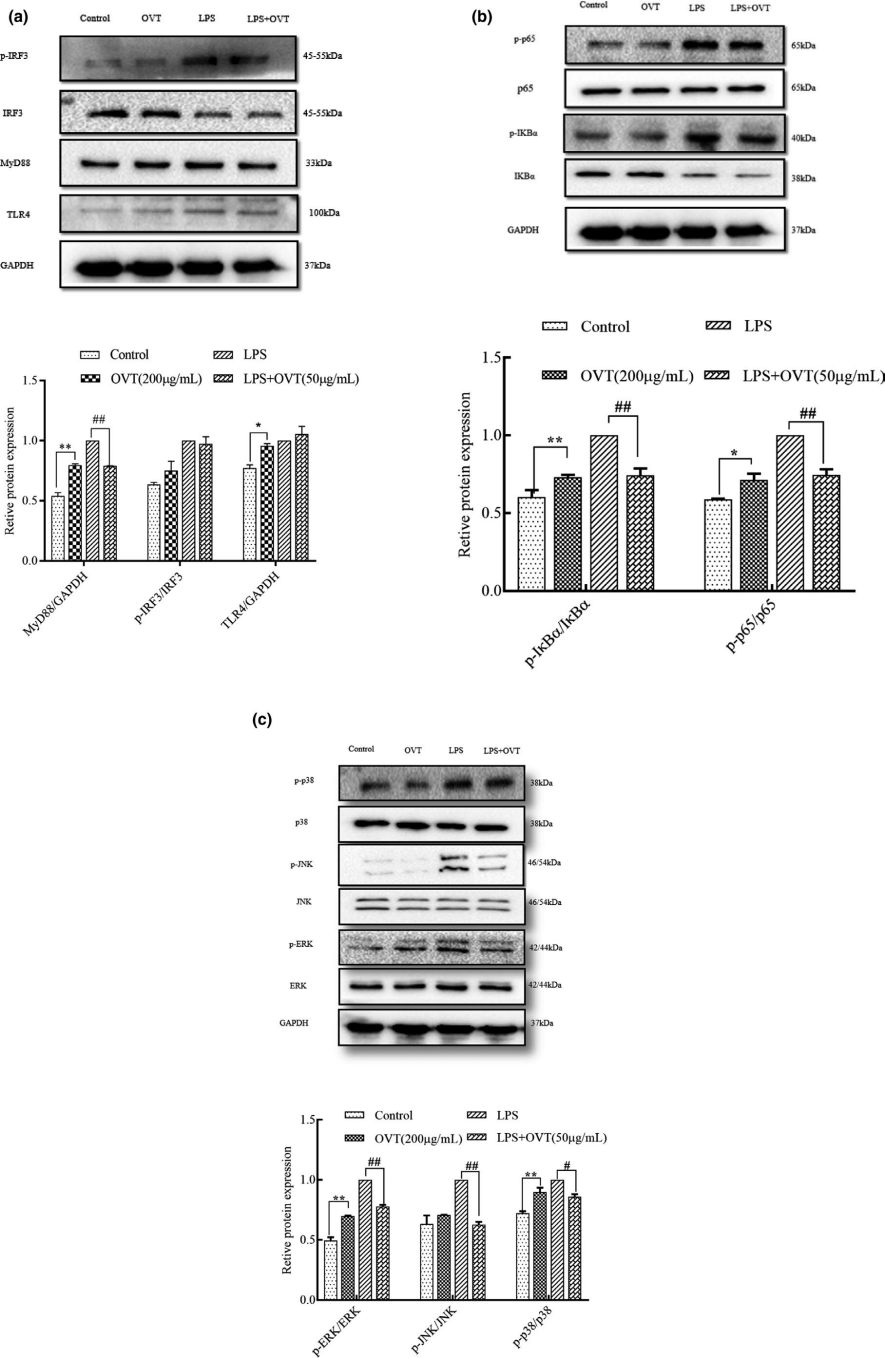
MyD88/NF‐κB /MAPK were involved in the immunity activity of OVT in RAW264.7 cells and LPS‐stimulated RAW264.7. Macrophages were incubated with various concentrations of OVT in the presence or absence of LPS (100 ng/ml) for 24 h. Cell lysates were immunoblotted for these protein with GAPDH used as control. (a) Expression of MyD88, IRF3, TLR4 and phosphorylation of IRF3. (b) IκBα, p65, phosphorylation of IκBα and p65. (c) MAPK phosphorylation. Results are shown as means ± *SEM* (*n* = 3). (a) Compared with the control group: *p < .05, **p < .01; (b) compared with the LPS group: ^#^ *p* < .05, ^##^ *p* < .01

In order to confirm whether MyD88, IRF3, NF‐κB and MAPK are involved in the immunity activity of OVT in RAW264.7, western blotting must be employed. It was clear (shown in Figure 8a,b) that expression of MyD88 protein, TLR4, phosphorylation of IκBα and p65 were increased by OVT (200 µg/ml) in RAW264.7 cells (*p* < .05). In addition, OVT significantly reduced expression of MyD88, TLR4, phosphorylation of IκBα and p65 in LPS‐stimulated RAW 264.7(*p* < .01). As shown in Figure 8a, compared with the control group, the level of IRF3 protein in the LPS‐stimulated group was decreased. However, following treatment with OVT, the expression levels of the phosphorylated proteins found no effect compared with the LPS‑only group. No apparently differences in the expression of IRF3 in RAW264.7 cells were found between control group and OVT group (*p* > .05). As expected in Figure 8c, the phosphorylation of JNK, p38 and ERK1/2 was significantly increased in LPS‐stimulated RAW264.7 cells (*p* < .01). Phosphorylation of p38 and ERK of RAW264.7 cells treated with OVT was increased (*p* < .05), but OVT did not affect phosphorylation of JNK. Phosphorylation of ERK, JNK and p38 was significantly decreased by OVT in LPS‐stimulated RAW264.7 (*p* < .05).

### 3.9 Effect of TLR4 receptor inhibitor on NO and cytokine secretion in RAW264.7 cells

**FIGURE 9:**
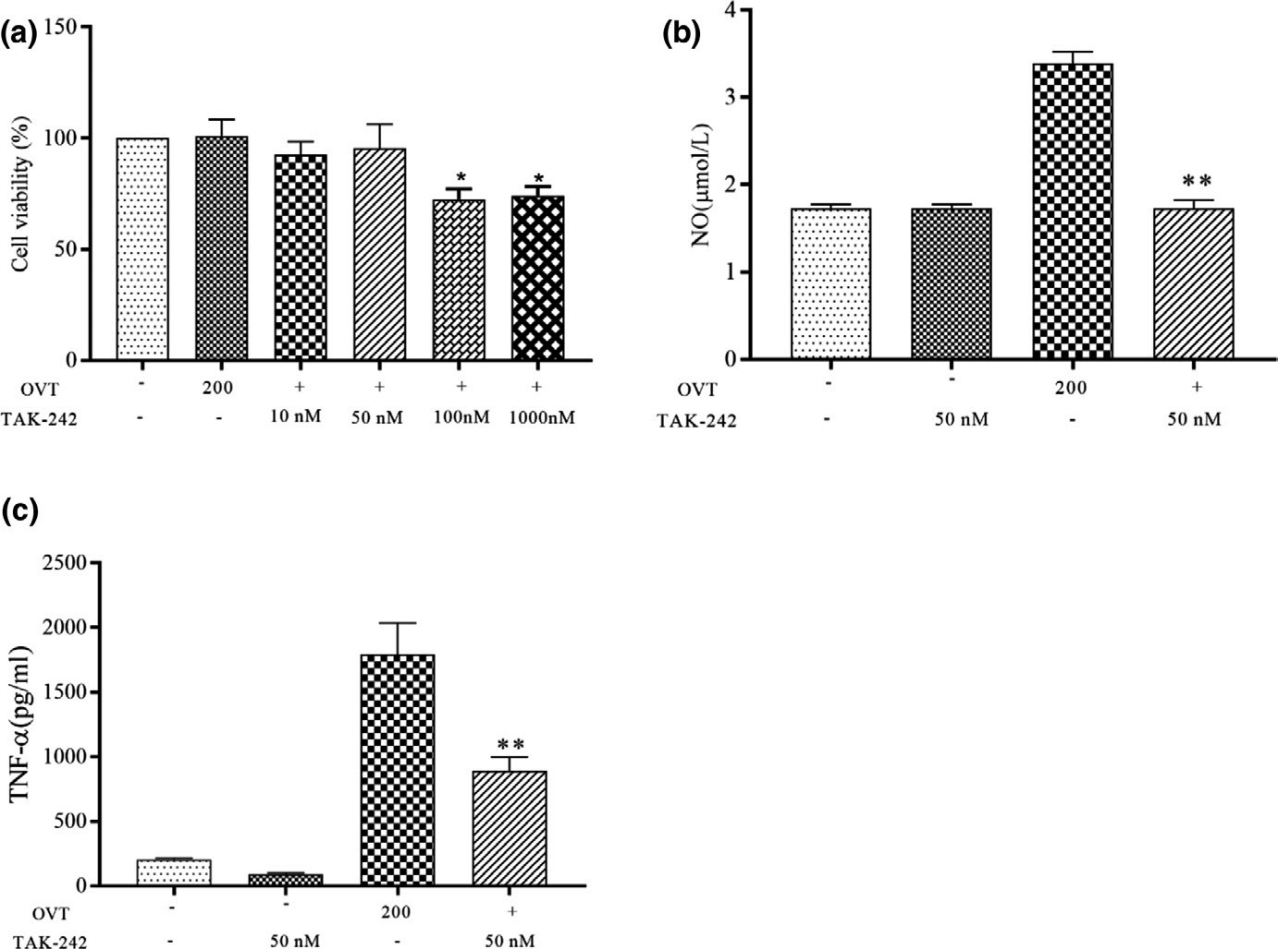
Effects of OVT on theTLR4 receptor. RAW264.7 cells were pretreated with or without the TLR4 inhibitor TAK‐242 for 8 h (a, b, c) After incubation with 200 μg/ml of OVT for 24 h, cell viability was determined by CCK, NO production was detected by Griess reagent, and TNF‐αproduction was detected by ELISA assay. *p < .05, **p < .01 compared with OVT

To determine whether OVT can induce the release of related cytokines through activation of TLR4 receptors, RAW264.7 macrophages were pretreated with a TLR4 inhibitor (Wen et al., 2019). Figure 9a shows the effect of different concentrations of TLR4 on cell viability at the concentration of blocker. We found that the 50 nM TAK‐242 blocker had no effect on cell viability, which could be used for subsequent experiments. The results showed in Figure 9b,c that 200 μg/ml OVT significantly increased the secretion of NO and TNF‐α, which were 3.8 µM and 1800 pg/ml, respectively. The levels of NO and TNF‐α in RAW264.7 macrophages treated with TLR4 inhibitor were significantly reduced than that of OVT group alone (*p* < .05).

## 4 DISCUSSION

Macrophages, as a group of mononuclear phagocytes, are widely found in the body and are critical in host defense due to coordination innate immunity and inflammatory responses. Inflammation is the immune defense response of the body for the purpose of self‐protection (Mosher et al., 2001). It is a common pathological process of the human body. It is closely related to atherosclerosis, asthma, rheumatoid arthritis, diabetes and other diseases. Inflammatory immune response at the damaged part of the body is conducive to pathogen removal, tissue cell regeneration and functional repair. RAW264.7 macrophage is the most‐used model about immune regulation of protein in vitro (Elisia et al., 2018; Saisavoey et al., 2020). When body receives external stimulation, macrophages firstly devour and clear foreign pathogens. Macrophages can swallow tumor cells and damaged cells, reflecting the immune function of proteins. In this study, macrophage phagocytosis by OVT significantly was enhanced, possibly because of the activation of TLR4 on the macrophage surface.

If proinflammatory cytokines are released in large quantities and inflammatory response is too strong, it will lead to increased tissue damage and induce serious immune damage of the body. Therefore, moderate inflammatory immune response is conducive to the body to remove pathogens and achieve self‐repair; once the inflammatory immune response exceeds a certain range, it will cause immune damage and harm to the body health (Laskin & Pendino, 1995). Cytokines and inflammatory mediators were also secreted by activated macrophage in the host defense of the immune system. It has been reported in the literature that *yupingfeng* fermentation polysaccharide promotes the expression of inflammatory cytokines in noninflammatory models on the one hand, and inhibits the expression of inflammatory cytokines in inflammatory models on the other hand (Sun et al., 2017). A growing body of support suggests that activated RAW264.7 macrophages can secrete some inflammation cytokines. Among them, TNF‐α and IL‐6 are representative major factors which often used to measure the severity of inflammation (Wang et al., 2014). When the inflammatory response reaches a certain level, IL‐10 is secreted to suppress inflammation. In our present study, OVT showed immunoregulatory activity, with NO production of LPS‐induced RAW264.7 treated with low concentration of OVT being inhibited, and increased secretion of the NO in a dose‐dependent manner in OVT‐induced RAW264.7 cells. These observations are in accordance with the previous finding that food‐derived protein anti‐inflammatory extracts markedly inhibit the release of NO in LPS‐stimulated RAW264.7 macrophages (Kim et al., 2020; Tagashira et al., 2018). Kim et al. (2020) found that the production of NO is inhibited by ovalbumin hydrolysate through reducing the phosphorylation of JNK and ERK in LPS‐stimulated RAW264.7. It is speculated that the possible reason is that OVT can control the NO release process by MAPK signaling pathway. Furthermore, the result of the study also showed that secretion and mRNA expression of IL‐6, TNF‐α and IL‐10 in RAW264.7 were promoted, which were part consistent with the previous reporting that OVT stimulated the release of IL‐6 from HD11 macrophages (Xie et al., 2002). And secretion and expression of the IL‐6, TNF‐α and IL‐10 were inhibited by OVT in LPS‐induced RAW264.7 cells. TNF‐α, as main upstream cytokines, induces the activation of the NF‐κB pathway and stimulates production and expression of pro‐inflammatory factors like IL‐6 (Li, Chang, et al., 2019). Fifty microgram per milliliter OVT suppressed level of IL‐10 in LPS‐stimulated RAW264.7 macrophages. This effect might be due to the significant reduction of TNF‐α and IL‐6 in LPS‐stimulated RAW264.7 cultured with 50 µg/ml OVT (Meram & Wu, 2017). Anti‐inflammatory activity of phosphopeptides (PPPs) from hen egg yolk phosvitin is reflected in inhibitory release of cytokine expression (Xu, Yang, et al., 2012). In summary, these results indicate that OVT has the ability to activate macrophages to secrete inflammatory cytokines and inflammatory mediators, and it is particularly important to study its mechanism of action.

The immune‐related receptors (PRR) on the cell surface are activated by foreign pathogen stimulation and initiate a series of tight signal cascades in the cells. The toll‐like receptor family (TLRs) is the most widely studied PRR. Over the past few years, it has been bound to a variety of ligands including sugars (Chen et al., 2019), proteins (Ren et al., 2010), and lipids (Diao et al., 2019). TLR4 is associated with LPS‐induced inflammation and is widely distributed in a variety of cells such as macrophages, monocytes, neutrophils and dendritic cells. Recently, a large number of studies have found that the protein may regulate the activity of immune cells by binding to receptors on cells. More importantly, CD14 is essential for TLR4/LPS recognition and TLR4 signaling triggering in cells stimulated with LPS. Thence, we attempt to determine whether an increase or decrease in the expression of CD14 and TLR4 in macrophages. In fact, in the current study, significant aggrandizement of TLR4 gene and protein expression was unexpectedly initiated in OVT‐treated RAW264.7 cells. From this point of view, it is of interest that TLR4 and CD14 mRNA expression were not significantly up‐regulated by OVT in LPS‐induced inflammatory models (Prymas et al., 2020), and Guo et al. clarified that κ‐Carrageenan hexamer worked to inhibit CD14 in LPS‐stimulated RAW264.7 and have anti‐inflammatory effect (Guo et al., 2019). Similar with study of Ganoderma protein receptor (Li, Chang, et al., 2019), the data showed that OVT may combine with TLR4 to exert immune enhancement functions, but it did not exert anti‐inflammatory function by inhibiting the expression of these two genes according the current observations (Lee et al., 2019). This also explains why OVT can significantly enhance the phagocytosis of macrophages to effectively eliminate potential pathogens. Lactoferrin suppresses LPS‐induced expression of NF‐кB pathway in RAW264.7 cells through TLR4 (Nemati et al., 2020), similar with which, inflammatory cytokines secreted by macrophages induced by OVT were down‐regulated, when cells were pretreated with inhibitors of TLR4. It indicated that OVT activated macrophages through TLR4 receptors to initiate downstream signaling pathways. Whey proteolytes treated with high pressure inhibited the secretion of TNF‐α and IL‐8 in LPS‐induced respiratory epithelial cells, but had no effect on the secretion of IL‐8 in TNF‐α and IL‐1‐induced cells, suggesting that whey proteolytes may reduce the expression of pro‐inflammatory factors through toll‐like receptor pathways. On the other hand, the hydrolysates did not down‐regulate TLR4 receptor expression, suggesting that whey protein may play a role in regulating inflammation by inhibiting LPS binding to toll‐like receptors and inhibiting NF‐κB signaling pathway activation, thereby regulating cytokine secretion (Iskandar et al., 2013).

MyD88 independent pathway, one of TLR4‐mediated signaling pathways, activates NF‐κB and MAPK. Interferon regulator 3 (IRF3) which has been demonstrated to regulate cells proliferation, apoptosis, inflammation, innate immune responses and insulin resistance was activated by TIR‐domain‐containing adaptor inducing interferon‐β (TRIF; Tong et al., 2020) in responses to LPS challenge. These cascade transcriptional responses induce stable expression of thousands of genes and ultimately regulate the release of inflammatory cytokines and anti‐inflammatory factors. It was found that the expression of MyD88 protein was up‐regulated in OVT‐induced cells and was effectively down‐regulated in LPS‐induced RAW264.7 cells. Additionally, we found that there were no significant changes in the phosphorylation levels of IRF3 protein in LPS‐stimulated macrophages treated with OVT, as compared with LPS‐stimulated cells. Comparison of control and OVT groups presented similar results. We proposed that OVT might play an immunomodulatory role through the MyD88 signaling pathway mediated by TLR4. Our results were similar to previous research results of wheat germ globulin (Wu et al., 2017) and soyasaponins (Chen et al., 2020).

As we all know, in the presence of external stimuli such as LPS, NF‐κB as a nuclear factor is responsible for regulating immune responses through regulating gene transcription in RAW264.7 cells (Li, Ye, et al., 2019). IκBα is degraded by phosphorylation and dissociated from the trimer, exposing the p65/p50 dimer. The dimer enters the nucleus from the cytoplasm through the translocation signal on the p65 subunit, thereby exerting a transcriptional regulatory role (Ren et al., 2020). In this study, the OVT group promoted the phosphorylation of IκB and p65 in macrophages and LPS‐induced cells were inhibited the phosphorylation of IκB and p65. MAPKs including the p38, ERK1/2 and JNK subgroups can regulate cell functions such as cell growth and gene expression as well as also plays a key role in the transcriptional activation of NF‐κB (Wang et al., 2018). Recently, OVT activates RAW264.7 macrophages through the MAPK pathway, promoting NO secretion, cytokine expression and phagocytic activity, and has immune enhancing activity (Lee et al., 2018). However, OVT is shown to significantly increase the phosphorylation of p38 and ERK, except that of JNK in our research. The one possibility is that the concentration of OVT may be relatively low, leading to an insignificant change in JNK. When OVT was added to LPS‐induced macrophages, the phosphorylation levels of JNK, ERK and p38 were significantly inhibited. These findings suggest that NF‐κB and MAPK signaling cascades both contribute to OVT related the process of immune regulation.

In conclusion, our study demonstrated immunomodulatory activities of OVT in RAW264.7 in vitro. The study indicates that high concentration of OVT promotes the accumulation of inflammatory mediator in RAW264.7. TLR4 was demonstrated to be a membrane receptor by which OVT may act on RAW264.7 cells, and the MAPK and NF‐κB signaling pathways were involved in OVT‐induced macrophage immunoregulation. Besides, low concentration of OVT suppressed LPS‐induced NO, TNF‐α and IL‐6 production by inhibiting NF‐κB and MAPK associated protein expression. Following our experimental results, OVT could be researched and developed as one of the potentially functional foods for immunological reagents.

## CONFLICT OF INTEREST

The authors declare that they do not have any conflict of interest.

## AUTHOR CONTRIBUTIONS

**Huaying Du:** Funding acquisition (supporting); Supervision (equal). **Zhiying Ru:** Data curation (equal); Formal analysis (equal); Writing‐original draft (equal). **Mingsheng Xu:** Conceptualization (equal); Writing‐review & editing (equal). **Gaoxiang Zhu:** Writing‐review & editing (equal). **Yonggang Tu:** Writing‐review & editing (equal). **Yan Jiang:** Methodology (equal).

## ETHICAL APPROVAL

This study does not involve any human or animal testing.

## Data Availability

The data that support the findings of this study are available from the corresponding author upon reasonable request.
